# Implicit Theory of Mind across the life span – Anticipatory looking data

**DOI:** 10.1016/j.dib.2017.10.021

**Published:** 2017-10-18

**Authors:** Louisa Kulke, Mirjam Reiß, Horst Krist, Hannes Rakoczy

**Affiliations:** a Department of Developmental Psychology, Institute of Psychology, University of Göttingen, Germany; b Department of Developmental Psychology and Educational Psychology, Institute of Psychology, University of Greifswald, Germany

## Abstract

In this work, we present a collection of data from three replication studies of anticipatory looking false belief tasks measuring implicit Theory of Mind. Two paradigms, by Southgate & Senju and Surian & Geraci were replicated in two independent labs. Eye-tracking data was collected and processed in line with the original procedures to allow for an investigation of effects of false belief processing on looking times and first saccades.

**Specifications table**

**Table:** 

Subject area	*Psychology*
More specific subject area	*Developmental Psychology*
Type of data	*Datasets containing processed eye-tracking data*
How data was acquired	*For dataset 1 gaze was recorded using a Tobii T120 eye tracker (Stockholm, Sweden ) at 60 Hz and analyzed using the Tobii Fixation Filter with default parameters (velocity and distance threshold: 35 pixels/samples).*
	*For datasets 2 and 3, an SMI REDn Scientific remote eye tracker recorded gaze positions at a rate of 60 Hz. Gaze information was saved for offline analysis, which was conducted using BeGaze Software (*version 3.5.101*), RStudio Version 0.99.893.*
Data format	*Processed using the algorithms based on the original studies that were replicated in this project*
Experimental factors	*The factors manipulated in the original paradigms: The belief version (FB1/FB2) in the Southgate/Senju paradigm was manipulated between participants and the Surian & Geraci conditions (TB/FB) were manipulated within participants, as in the original studies respectively.*
Experimental features	*Participants were seated in front of a monitor of the eye-tracking system (dataset 1) or a laptop with the remote eye tracker attached to it (datasets 2 and 3). The participants’ distance and angle to the screen were adjusted to the height of the participant to ensure the best possible eye-tracking signal.*
	*For dataset 1, participants viewed either the FB1 or the FB2 condition of a reconstructed version of the Southgate/Senju paradigm. Examples of the videos employed can be viewed at*https://youtu.be/21dII2jlpxw*,*https://youtu.be/f7YtiTCjRWE*, and*https://youtu.be/1pkNA2zzIrQ*, for a familiarization, FB1, and FB2 trial, respectively.*
	*For dataset 2, participants viewed either the FB1 or the FB2 condition of the original version of the Southgate/Senju paradigm.*
	*For dataset 3, participants were presented with two paradigms (Southgate/Senju and Surian & Geraci) in a randomized order. Each paradigm was preceded by a 5 point calibration and validation routine. The Southgate/Senju condition (FB1 or FB2) was randomly selected for each participant. Participants received both a TB and an FB condition in the Surian & Geraci paradigm, however, the order of the test trials was randomized and the screen side (left, right) was counterbalanced. The testing procedure took approximately 10 min.*
Data source location	*University of Greifswald, Greifswald, Germany (dataset 1)*
	*University of Göttingen, Göttingen, Germany (datasets 2 and 3)*
Data accessibility	*The data is published alongside this article*
Related research article	*Louisa Kulke, Mirjam Reiß, Horst Krist, & Hannes Rakoczy (inpress). How robust are anticipatory looking measures of Theory of Mind? Replication attempts across the life span. Cognitive Development.*https://doi.org/10.1016/j.cogdev.2017.09.001.

**Value of the data**

• The current datasets provide data from independent replications of anticipatory looking implicit Theory of Mind tasks.
• This allows for an investigation of the replicability of implicit Theory of Mind tasks.
• As several paradigms were completed by each subject in one session, the data allow for an investigation of convergent validity of these paradigms.
• As different age groups were tested, the dataset allows for an investigation of changes in implicit Theory of Mind across the life span.

## 1 Data

Dataset 1: The dataset contains the differential looking score (DLS) of looking times to areas of interest (AOIs), the first saccades to AOIs, first looks to AOIs as well as raw looking times to the correct and incorrect AOIs. Factors include SubjectID, Sex, AgeGroup, Age in months, condition (1 = FB1, 2 = FB2), Order of Presentation (1 = LRRL, 2 = RLLR), Turning Direction of the actress (1 = counterclockwise 1st, 2 = clockwise 1st), inclusion according to the Senju criterion and the Southgate criterion (0 = no, 1 = yes) and the verbal answer (0 = incorrect, 1 = correct, 2 = other).

Dataset 2 contains the DLS, the first saccades to AOIs and first looks to AOIs. Factors include SubjectID, Age in months, inclusion according to the Senju criterion (FamiliarizationCorrect) and the condition (FB1 or FB2).

Dataset 3: The dataset contains the differential looking score of looking times to AOIs, the first saccades to AOIs for the Surian & Geraci and the Southgate/Senju paradigm and the raw looking times for the Surian & Geraci paradigm, which are the outcome measures used in the original studies respectively. Factors include FamiliarisationOK for the paradigms, indicating whether subjects passed inclusion criteria for the familiarization (1) or not (0), Age group (1 = Child, 2 = Adult, 3 = Elderly Adult), Southgate/Senju FB Version (Stimulus FB1 or FB2) Belief (True or False (TB/FB)), Location (location of the AOI, defined as either belief-congruent or belief-incongruent or defined as containing the object of interest or not). The variable SubjectNo makes it possible to include the subjects into analyses; in order to anonymize the dataset, numbers were assigned randomly to each subject.

## 2 Experimental design, materials and methods

The current datasets can be used, firstly, to test for the robustness of implicit ToM tasks across the life span and, secondly, to investigate the convergent validity of two main implicit ToM tasks.

## 3 Dataset 1

### 3.1 Participants

For this dataset, 495 participants were recruited. Of these, 35 children had to be excluded because of missing looking data (*n* = 21) or other technical issues (*n* = 2), discontinuation of the experimental session (*n* = 6), experimenter error (*n* = 1), noncompliance (*n* = 2), or a known mental retardation (*n* = 3). The remaining 460 children were divided into five age groups: There were 116 two-year-olds (44 female, *M* = 29.5 months, SD = 3.5, range = 24–35 months); 88 three-year-olds (45 female, *M* = 42.0 months, SD = 3.5, range = 36–47 months); 90 four-year-olds (38 female, *M* = 54.1 months, SD = 3.1, range = 48–59 months); 83 five-year-olds (43 female, *M* = 65.2 months, SD = 3.3, range = 60–71 months); and 83 six-year-olds (44 female, *M* = 76.6 months, SD = 3.3, range = 72–83 months). All children lived in or close to the city of Greifswald, located in the Northeast of Germany. They participated on a voluntary basis and with the consent of their parents. Children were rewarded with small presents and parents were compensated for travel costs.

### 3.2 Stimuli

Like in the original studies by Southgate and her collaborators [1], [2], familiarization and test events were presented as video clips and children's looking behavior was registered via an eye tracker. All videos were recorded following the scripts for the original videos employed by Southgate et al. [2]. A female actor stood behind a panel containing two windows, and in front of each window there was an opaque box with a lid. At the start of each trial, a puppet appeared at the center of the stage and placed a colored ball in one of the boxes. The actor wore a visor covering her eyes to eliminate the possibility of using eye gaze as a cue to where she would search. She did, however, always follow the puppet's movements with her head.

In four familiarization trials, children observed how the puppet opened the lid of the left- or right-hand box, put the ball into it, closed the lid, and then disappeared. After this, the actor retrieved the ball by reaching through the corresponding window on the left- or right-hand side. Before the window began to open, both windows were illuminated (for 1 s) and a chime sounded simultaneously to signal the forthcoming reaching action (2.75 s after the onset of illumination). Familiarization trials were presented in two different orders with respect to the placement of the ball: (a) left - right - right - left, or (b) right - left - left - right. After each familiarization trial an attention getter (i.e., a jiggling and sounding toy animation) appeared in the center of the computer screen.

Following familiarization, one of two false-belief test trials (FB1 or FB2) was presented. In the FB1 condition, the puppet opened the lid of the left-hand box, put the ball into it, closed the lid, returned to the center, retrieved the ball again, placed it in the center of the stage, opened the right-hand box, put the ball into it, closed the right- and the left-hand-box (in this order), and then disappeared from the scene. Then the sound of a phone ringing was heard, whereupon the actor turned away from the stage as if she was attending to the sound. As soon as the actor turned around, the puppet returned, retrieved the ball from the box, closed the lid, and disappeared from the scene. Once the phone stopped ringing, the actor turned back, the windows were illuminated, and the chime sounded.

In the FB2 condition, the puppet also put the ball into the left-hand box but disappeared from the scene right after this and before the phone was ringing and the actor turned around. Then the puppet returned, retrieved the ball from the left-hand box, placed it in the center of the stage, opened the right-hand box, put the ball into it, closed the right- and then the left-hand box, paused shortly, opened the right-hand-box, retrieved the ball, closed the box, and disappeared from the scene. After this the phone stopped ringing, the actor turned back, the windows were illuminated, and the chime sounded. Each test video continued for 5 s after the onset of illumination of the windows without any more action.

In the original studies [1], [2], the actor's turning direction was confounded with the belief condition: In the FB1 condition, the actor turned around in a clockwise direction and back in a counter-clockwise direction, whereas, in the FB2 condition, she moved in the opposite direction, respectively. We eliminated this confound by using both types of turning movements in both belief conditions.

### 3.3 Design and distinctive features

Participants were randomly assigned (between subjects) to one of the eight conditions resulting from the combination of the experimental variable condition (FB1 vs. FB2) with the control variables order of presentation (of the familiarization trials) and turning direction. Age group was included as an additional variable in a full-factorial between-participant design.

The major deviations of the present data collection from those of Southgate et al. [2] and Senju et al. [1] can be summarized as follows: (1) While Southgate et al. [2] tested 24- and 25-month-old toddlers and Senju et al. [1] 6- to 8-year-old normally developing children (besides children with autism spectrum disorder), we recruited a large sample of toddlers and preschoolers (ranging from 2;0 to 5;11). (2) Like Senju et al. [1], but unlike Southgate et al. [2], we presented participants with four rather than two familiarization trials. (3) The order of the familiarization trials as well as the actor's turning direction were varied across participants.

### 3.4 Procedure

Children were tested individually by a female experimenter. They watched the video clips on a computer monitor (17” monitor, 1280 × 1024 pixels) while their eye movements where recorded using a Tobii T120 eye tracker (Stockholm, Sweden). The videos were presented using Tobii Studio 2.2.7 software. Eye gaze was registered at a sampling frequency of 60 Hz and analyzed using the Tobii Fixation Filter with default parameters (velocity and distance threshold: 35 pixels/samples). Children sat at a distance of approximately 60 cm from the monitor, either on their parent's lap or on a child's seat (Tripp Trapp, Stokke AS, Norway). The eye tracker was calibrated individually using the regular 5-point calibration procedure of Tobii Studio with default settings (red dots, gray background, medium speed, full screen). If necessary, the calibration procedure was repeated before the experimental session started with the familiarization trials. After the test trial, while the last frame of the scene was still visible, children were asked "Where do you think is she going to look for the ball first?"

### 3.5 Data processing

Children's anticipatory looking was analyzed in two ways: (1) by measuring their looking times to each window (during an interval of 5 s starting with the onset of illumination of the windows), and (2) by registering which of the two windows they fixated first (within an interval of 2 s after the onset of illumination of the windows). For this purpose, two rectangular areas of interest (AOIs) were defined covering each of the two windows. For the first measure, a differential looking score (DLS) was calculated as the difference between the looking time to the correct window and the incorrect window divided by the total looking time to both windows (no preference = 0, minimum = −1, maximum = 1):

$$\mathrm{DLS}=\frac{t(\text{corr)}-t(\mathrm{incorr})}{t(\mathrm{corr})+t(\mathrm{incorr})}$$

There were 8 children (3 two-year-olds, 2 five-year-olds, and 3 six-year-olds) whose anticipatory looking could not be analyzed because of missing data.

## 4 Dataset 2

### 4.1 Participants

An opportunity sample of *N* = 52 neurotypical children (*M* = 60.7 mos, SD = 25.75 mos, range = 24–127 mos, 34 female) participated at a family fair after parental consent was obtained. Children received a sticker in return for their participation.

### 4.2 Materials and stimuli

Children watched one of two original videos from Senju et al. [3] (between subjects variable condition FB1/FB2), which is comparable in structure to the paradigm described for dataset 1. In Senju et al. [3] videos an actress wearing a visor sits behind an occluder and reaches through one of two windows in the occluder to retrieve a toy. In the first two familiarization trials this toy is lying on one of two boxes. In the third and fourth familiarization trial a handpuppet puts the toy in one of the two boxes. Subjects are then presented with one of two test trials in which the puppet puts the toy in a box and then moves it to the other box either while the actress was watching (FB1) or not (FB2). In both trials the puppet then removes the toy from the scene without the actress watching; therefore, the actress has a false belief about the location of the object in both trials.

A remote SMI REDn eye tracker was used to monitor participants’ gaze. Before recording started, participants completed a standard 5 point calibration and validation routine. Movies were controlled in the SMI Experiment Center (version 3.5.169) and presented on a 15.6 in. LCD screen (1920 × 1080 pixel). Gaze information was saved for offline analysis, which was conducted using BeGaze Software (version 3.5.101) for AOI processing.

### 4.3 Procedure

Participants were seated in front of a laptop with the eye tracker attached to it and a 5 point calibration and validation was obtained. The testing procedure took approximately 3 min. The condition was randomly assigned to each participant.

### 4.4 Analysis

Based on the original analysis procedure [3], the first saccade after the chime sound and fixations to both windows in the occluder were measured during the freeze period and a DLS was calculated as described for dataset 1.

## 5 Dataset 3

In a within-subject design, two main change-of-location false belief (FB) paradigms were used and tested between-subjects in children, adults and elderly adults.

### 5.1 Participants

Sixty-four neurotypical children (*M* = 5.01 years, SD = 0.392, range = 4.5–5.5 years), 80 neurotypical adults (*M* = 24.07 years, SD = 2.952) and 83 elderly adults (*M* = 71.43 years, SD = 6.953) volunteered to participate. All participants had normal or corrected to normal vision and reports of known visual symptoms (e.g., cataracts in elderly participants) were noted. Participants who did not pass inclusion criteria based on original studies can be excluded from further analyses, leading to a total participant number of 40 children, 40 adults and 40 elderly adults. Consent was obtained from adult participants and from the children's parents. The study was approved by the ethics committee of Göttingen University (Ethics code: 143a).

### 5.2 Materials and stimuli

The original stimuli from Senju et al. [3] and Surian and Geraci [4] were provided by the authors.^1^ The Senju et al. videos were identical to the original videos described for dataset 2.

Surian and Geraci [4] videos show a triangle chasing a ball through a Y-shaped tunnel. In two familiarization trials the triangle chases the ball until it enters the Y-shaped tunnel from the bottom end. It then waits until the ball reappears (either at the left or right top end) and hides in a box at that end. The triangle then moves into the tunnel and reappears at the same end as the ball. The two test trials are identical to the familiarization trials up to the point where the ball enters the box. At this point the triangle either leaves the scene, the ball moves from the box at one end of the tunnel to the box at the other end and the triangle then reappears to the scene (false belief condition) or the triangle witnesses that the ball moved to the other box, then disappears and reappears (true belief condition). The triangle then enters the tunnel and reappears at the belief-congruent location.

### 5.3 Equipment

An SMI REDn Scientific remote eye tracker was used to monitor participants’ gaze. Before recording started, participants completed a standard 5 point calibration and validation routine. Movies were presented on a laptop computer (DELL Precision M4800 with a Windows 8.1 pro operating system) controlled via the SMI Experiment Center (version 3.5.169) and presented on a 15.6” LCD display (1920 × 1080 pixel). Gaze information was recorded at 60 Hz and saved for offline analysis, which was conducted using BeGaze Software (version 3.5.101) for data export.

### 5.4 Design

In a mixed design, all participants completed the Southgate/Senju and the Surian & Geraci task. The belief version (FB1/FB2) in the Southgate/Senju paradigm was manipulated between participants and the Surian & Geraci conditions (TB/FB) were compared within participants, as in the original studies respectively. The original outcome measures were adopted in the current data set; therefore, first saccades and DLS are reported for the Southgate/Senju paradigm and first saccades and proportion of looking times for Surian & Geraci. However, DLS was computed to allow for analysis of correlations of the different paradigms as it is independent of the time window chosen for looking time analysis.

### 5.5 Procedure

Participants were seated in front of a laptop with the remote eye tracker attached to it. The participants’ distance and angle to the screen were adjusted to the height of the participant to ensure the best possible eye-tracking signal. Participants were presented with both paradigms in a randomized order. Each paradigm was preceded by a 5 point calibration and validation routine. The Southgate/Senju condition (FB1 or FB2) was randomly selected for each participant. Participants received both a TB and an FB condition in the Surian & Geraci paradigm, however, the order of the test trials was randomized and the screen side (left, right) was counterbalanced. The testing procedure took approximately 10 min.

## 6 Data processing

It was determined whether participants would be included in the analysis based on the inclusion criteria defined by Senju et al. [3] (longer looking towards the correct than the incorrect box in the last familiarization trial) and by Surian and Geraci [4] (longer looking towards the correct than towards the incorrect box in at least one of the two familiarization trials) and this was coded in the variables “Familiarisation”.

Measurements of looking times towards the AOIs were based on the original studies. In the Senju et al. [3] paradigm, looking times at the windows in the occluder were determined. In Surian and Geraci [4] paradigm, looking times towards an area including the boxes in the time window between the triangle entering the tunnel and its reappearance were measured. For the Surian & Geraci paradigm, looking times were the main measure, here depicted as the proportion of looking time on the AOIs during the defined time window. As the main Senju/Southgate measure, a differential looking score (DLS) was calculated as the difference in looking time to the correct minus the incorrect AOI divided by the overall looking time to both AOIs as described above. This DLS can furthermore be used for correlation analyses of the different paradigms as it is comparable and accounts for the different time windows defined for the AOIs.

To account for the true belief (TB) control condition in the Surian & Geraci's paradigm, the differential score described above was calculated for the FB and the TB condition and averaged to get an overall measure of belief-congruent looking (composite looking score, CLS).

$$\mathrm{composite looking score}=\frac{\frac{t(\mathrm{FB}\text{corr)}-t(\mathrm{FBincorr})}{t(\mathrm{FBcorr})+t(\mathrm{FBincorr})}+\frac{t\left(\mathrm{TBcorr}\right)-t(\mathrm{TBin}\text{corr)}}{t(\mathrm{TBcorr})+t(\mathrm{TBincorr})}}{2}$$

with *t*() being the time spent looking at each location, FB indicating the FB trials, TB indicating TB trials and corr being the correct and incorr the incorrect location. The DLS can vary between −1 (complete failure to look at the belief-congruent side in both TB and FB conditions) and 1 (perfectly belief-congruent gaze pattern in TB and FB conditions), with zero indicating that subjects do not look longer at one side (belief-congruent or –incongruent) on average.

## Footnotes

1 Note that of the Surian & Geraci paradigm only the original FB and TB videos for one object location were available from the authors. Therefore, in order to randomize the object location (left/right), as described in the original study, we mirrored the original videos at the central vertical axis to create identical videos with the object location on the respective other side.
